# The value of machine learning based on CT radiomics in the preoperative identification of peripheral nerve invasion in colorectal cancer: a two-center study

**DOI:** 10.1186/s13244-024-01664-1

**Published:** 2024-04-05

**Authors:** Nian-jun Liu, Mao-sen Liu, Wei Tian, Ya-nan Zhai, Wei-long Lv, Tong Wang, Shun-Lin Guo

**Affiliations:** 1 The First School of Clinical Medical, Lanzhou University, LanzhouGansu, 73000 China; 2 Department of Radiology, the First Hospital of Lanzhou University, LanzhouGansu, 73000 China; 3 Intelligent Imaging Medical Engineering Research Center of Gansu Province, LanzhouGansu, 73000 China; 4 Accurate Image Collaborative Innovation International Science and Technology Cooperation Base of Gansu Province, LanzhouGansu, 73000 China; 5 Gansu Province clinical research center for radiology imaging, LanzhouGansu, 73000 China; 6 Lichuan People’s Hospital, Lichuan, 445400 Hubei China

**Keywords:** Colorectal cancer, Computed tomography, Machine learning, Perineural invasion, Radiomics

## Abstract

**Background:**

We aimed to explore the application value of various machine learning (ML) algorithms based on multicenter CT radiomics in identifying peripheral nerve invasion (PNI) of colorectal cancer (CRC).

**Methods:**

A total of 268 patients with colorectal cancer who underwent CT examination in two hospitals from January 2016 to December 2022 were considered. Imaging and clinicopathological data were collected through the Picture Archiving and Communication System (PACS). The Feature Explorer software (FAE) was used to identify the peripheral nerve invasion of colorectal patients in center 1, and the best feature selection and classification channels were selected. Finally, the best feature selection and classifier pipeline were verified in center 2.

**Results:**

The six-feature models using RFE feature selection and GP classifier had the highest AUC values, which were 0.610, 0.699, and 0.640, respectively. FAE generated a more concise model based on one feature (wavelet-HLL-glszm-LargeAreaHighGrayLevelEmphasis) and achieved AUC values of 0.614 and 0.663 on the validation and test sets, respectively, using the “one standard error” rule. Using ANOVA feature selection, the GP classifier had the best AUC value in a one-feature model, with AUC values of 0.611, 0.663, and 0.643 on the validation, internal test, and external test sets, respectively. Similarly, when using the “one standard error” rule, the model based on one feature (wave-let-HLL-glszm-LargeAreaHighGrayLevelEmphasis) achieved AUC values of 0.614 and 0.663 on the validation and test sets, respectively.

**Conclusions:**

Combining artificial intelligence and radiomics features is a promising approach for identifying peripheral nerve invasion in colorectal cancer. This innovative technique holds significant potential for clinical medicine, offering broader application prospects in the field.

**Critical relevance statement:**

The multi-channel ML method based on CT radiomics has a simple operation process and can be used to assist in the clinical screening of patients with CRC accompanied by PNI.

**Key points:**

• Multi-channel ML in the identification of peripheral nerve invasion in CRC.

• Multi-channel ML method based on CT-radiomics can detect the PNI of CRC.

• Early preoperative identification of PNI in CRC is helpful to improve the formulation of treatment strategies and the prognosis of patients.

**Graphical Abstract:**

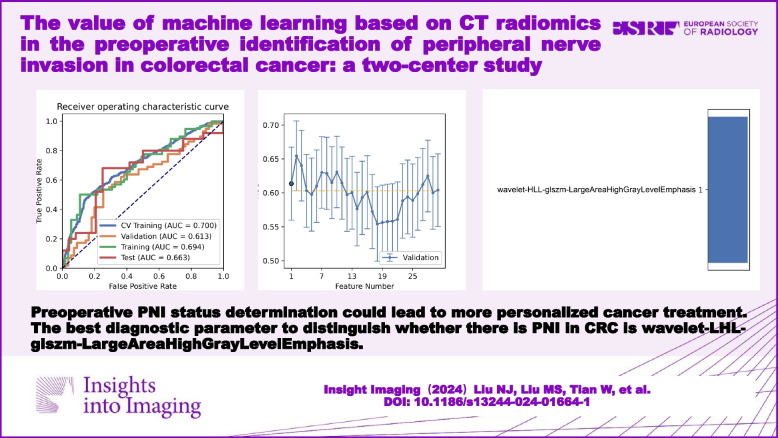

**Supplementary Information:**

The online version contains supplementary material available at 10.1186/s13244-024-01664-1.

## Introduction

In recent years, the global incidence and mortality rates of colorectal cancer (CRC) have significantly increased due to the impact of unhealthy lifestyle habits and an imbalanced dietary structure [1]. The Global Cancer Observatory (GLOBOCAN) project of the Global Cancer Research Center estimates that about 1.93 million cases of colorectal cancer were diagnosed worldwide in 2020, and about 930,000 people died of the disease [2]. In China, about 30% of CRC patients are diagnosed as middle to late-stage and are prone to peripheral nerve invasion (PNI) and distant metastasis. These patients are either unable to be surgically resected or have a poor prognosis after surgery, with local and distant recurrence rates remaining high, at about 4% and 15%, respectively [3]. The long-term survival rate of patients with CRC metastasis is far from our expectations [4].

Standardized and reasonable radical resection remains the preferred treatment for patients with early and middle-stage colorectal cancer. PNI is an underrated measure of character that the National Integrated Cancer Network lists as an important parameter that must be reported in standard pathology reports [5]. PNI means that the tumor invades the nerve structure or spreads along the nerve sheath [6, 7], which can adversely affect the prognosis of colorectal cancer [8, 9]. PNI may be a source of metastatic spread that can lead to poorer prognosis and reduced survival in colorectal cancer [6, 10–13]. Some studies have shown that the presence of PNI-positive CRC after surgical resection may indicate incomplete resection and local recurrence [11, 14]. Liebig et al. [14] reported that about 30–40% of patients with colorectal cancer have PNI positivity; the 5-year productivity after surgical resection is no more than 16 %, and the median survival time is 25 months. Some studies have shown that PNI-positive patients can benefit from adjuvant therapy. For PNI-positive CRC patients after local resection, it is usually recommended to expand the scope of surgical resection or receive adjuvant chemoradiotherapy [15–17]. Therefore, PNI status should be considered in stratified adjuvant therapy for colorectal cancer patients, and preoperative detection of PNI status has crucial clinical significance [13, 18].

PNI is primarily detected through pre-operative biopsy or post-operative histopathological examination [19, 20]. Biopsies are not effective in detecting PNI because they focus on the wrong layers and lack suitable specimens, hindering their use for diagnosing PNI status [21]. PNI status detection is limited in efficiency and timeliness, which restricts its application in guiding preoperative treatment decisions [19, 20, 22]. The National Comprehensive Cancer Network guidelines recommended magnetic resonance imaging (MRI) and computed tomography (CT) as the primary diagnostic methods for preoperative clinical staging of CRC, with traditional CT-enhanced scanning commonly utilized for tumor-lymph node-metastasis (TNM) staging and prognosis assessment[23]. MRI and CT are important methods for clinical evaluation of colorectal cancer. Unfortunately, neither can predict the PNI status of patients with colorectal cancer before surgery. Predicting the PNI status of CRC before treatment will help clinicians develop more personalized treatment strategies. Abdominal-enhanced CT is a cost-effective way to evaluate tumors in the medical field [1]. Previous studies on predicting PNI of colorectal cancer primarily used 2D region of interest (ROI) for lesion delineation [24–27]. 3D imaging model outperforms 2D imaging model in diagnosing gastrointestinal tumors [28, 29]. 3D radiomics models provide more comprehensive information on tumor heterogeneity and accurately reflect its biological behavior.

As far as we know, there is a lack of relevant studies on identifying PNI in CRC by CT radiomics ML method. As a branch of artificial intelligence, ML consists of multiple algorithms that analyze large and complex data sets to improve the results of neuro-oncology medicine in diagnosis, treatment, and follow-up [11]. ML is objective and repeatable, providing the best predictive power and clinical applications[30].

Therefore, this study aims to utilize ML algorithms based on radiomic features from whole-tumor CT images to identify the status of perineural invasion in colorectal cancer. By comparing 3 feature selection methods and10 ML algorithms in terms of their performance in identifying perineural invasion, the goal is to obtain the optimal pipeline for constructing a convenient, stable, and accurate ML prediction channel for patients undergoing colorectal cancer surgery.

## Materials and methods

### Patients

This retrospective study was conducted in two hospitals (The First Hospital of Lanzhou University, Gansu Province, Lanzhou, China; Lichuan People’s Hospital, Hubei Province. Lichuan, China). The ethics committee approved and waived the patient’s informed consent requirement.

A total of 268 consecutive patients with colorectal cancer who underwent CT examination at two hospitals between January 2016 and December 2022 were included in the study. Inclusion criteria are as follows: (1) patients with colorectal cancer confirmed by postoperative pathology; (2) complete clinical and pathological data and clear pathological report of PNI status; (3) enhanced CT scan was performed within 2 weeks before surgery, and the image quality met the requirements; (4) no other treatment was received before surgery. Exclusion criteria are as follows: (1) clinical and pathological data are incomplete, or lack complete preoperative CT images; (2) combined with other malignant tumors; (3) received radiotherapy, chemotherapy, or other treatments before surgery.

**Fig. 1:**
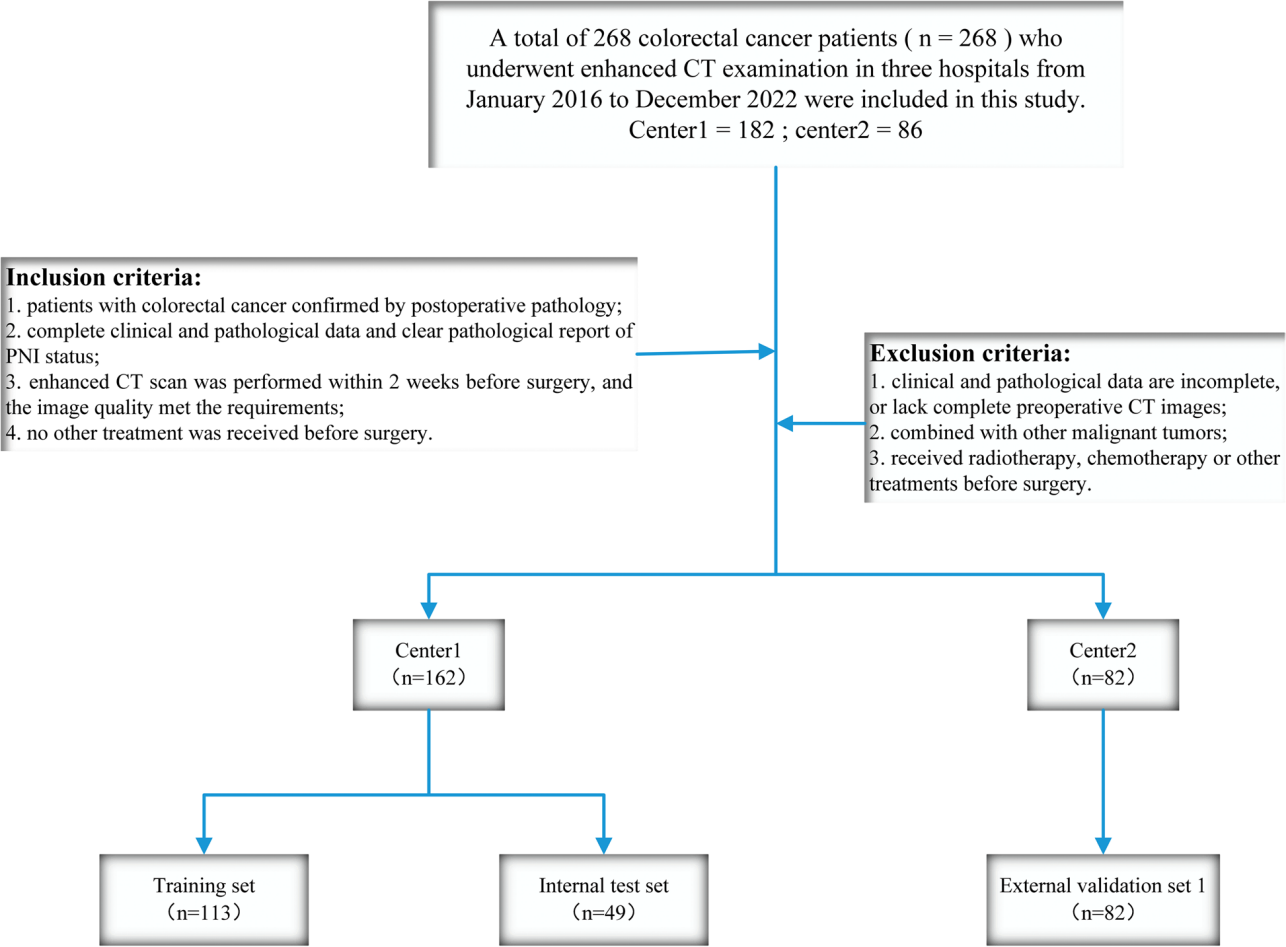
The inclusion and exclusion criteria of patients

Finally, we included 162 patients with colorectal cancer from hospital 1 (center 1) and 82 patients from hospital 2 (center 2). In center 1, the patients were randomly divided into a training set (*n* = 113) and an internal test set (*n* = 49) in a 7:3 ratio, while center 2 was used as an external test set (*n* = 82). Clinical data of each patient were obtained through the Picture Archiving and Communication System (PACS) (Fig. 1).

### Histopathologic standard for the determination of PNI

In this retrospective study, the PNI status of all CRC patients was obtained based on pathology reports of excised specimens. (1) At least 33% of the perinerve is surrounded by cancer cells (not invading the nerve sheath), and PNI status is defined as positive; (2) cancer cells are located in any layer of the nerve sheath [31, 32].

### Image acquisition and segmentation

The patient was placed supine and scanned from the top of the diaphragm to the symphysis pubis. Detailed CT scan parameters are shown in Supplementary Table A1. Since portal vein phase images are the best method for displaying colorectal cancer, we selected portal vein phase images as the target area. To eliminate potential differences in CT images obtained from different CT scanners at each center, before extracting radiomics features, the gray discrete method was used to normalize the final 256 bins of all original CT images (Analysis Kit software, version v3.0.0r, GE Healthcare) [32].

The conventional CT portal images were uploaded to the open-source software 3D Slicer 5.2.1 (https://download.slicer.org/). The whole tumor region of interest (ROI) was delineated by two doctors with 5 years (reader A, LIU Nian-jun) and 10 years (reader B, ZHAI Ya-nan) of abdominal lesion diagnosis experience, respectively, without knowledge of the specific lesion. Colorectal wall thickening or mass formation and significant enhancement were considered as potential tumor lesions, while the contents and gasses within the intestinal cavity were avoided during the delineation process as much as possible. After the delineation was completed, the ROI of all lesions was reviewed and calibrated by another physician (reader C, LV Wei-long), and any discrepancies were resolved through consensus among the three physicians. Additionally, intra-observer and inter-observer repeatability were evaluated by randomly selecting 30 patients and having reader A perform the delineation. The intraclass correlation coefficients (ICCs) within and between groups were all > 0.75, indicating good reproducibility and reliability of the features, which were subsequently used for further analysis.

### Radiomics extraction and selection

We utilized 3D-Slicer to extract radiomics features from each ROI. Prior to feature extraction, CT images were resampled for standardization. The extracted features in this study include First order (maximum, skewness, mean, median), GLCM (gray-level co-occurrence matrix), GLDM (gray-level dependence matrix), GLRLM (gray-level run length matrix), GLSZM (gray-level size zone matrix), NGTDM (gray-level run length matrix), and SHAPE. We selected robust features with an ICC > 0.75.

The Feature Explorer software (FAE, v0.5.5) is developed using the Python programming language (3.7.6) (https://github.com/salan668/FAE) [33]. Firstly, we utilized a computer-generated random dataset for the data in center 1. We assigned 70% of the dataset to the training set (*n* = 113) and the remaining 30% to the independent test set (*n* = 49). To address the imbalance in the training dataset, we duplicated random cases to achieve a balanced ratio between positive and negative samples. The dataset was normalized using *Z*-score normalization. Secondly, we employed the Pearson correlation coefficient (PCC) to measure the correlation between each pair of features and reduce the dimensionality of the feature matrix. If the PCC exceeded 0.99, one of the features was randomly removed. Finally, feature selection was performed using analysis of variance (ANOVA), Relief, and recursive feature elimination (RFE). Multivariate analysis of variance calculated the *F*-value weight of each feature with respect to the label, sorting them from largest to smallest to determine the most relevant features. The Relief algorithm assessed the correlation between features and categories based on their discriminative power among nearby samples. RFE, or recursive feature elimination, involved iteratively building a model (such as an SVM or regression model), selecting the best (or worst) features based on their coefficients, setting them aside, and repeating the process on the remaining features until all features were evaluated. We considered the number of features ranging from 1 to 30.

### Classifications

Ten machine learning algorithms were utilized to evaluate classification performance, based on Python code and the sci-kit learn library (https://scikit-learn.org/). These algorithms include support vector machines (SVM), linear discriminant analysis (LDA), AdaBoost (AB), Gaussian processes (GP), autoencoders (AE), random forests (RFE), logistic regression (LR), lasso logistic regression (LRLasso), decision trees (DT), and naive Bayes (NB).

### Evaluations

**Fig. 2:**
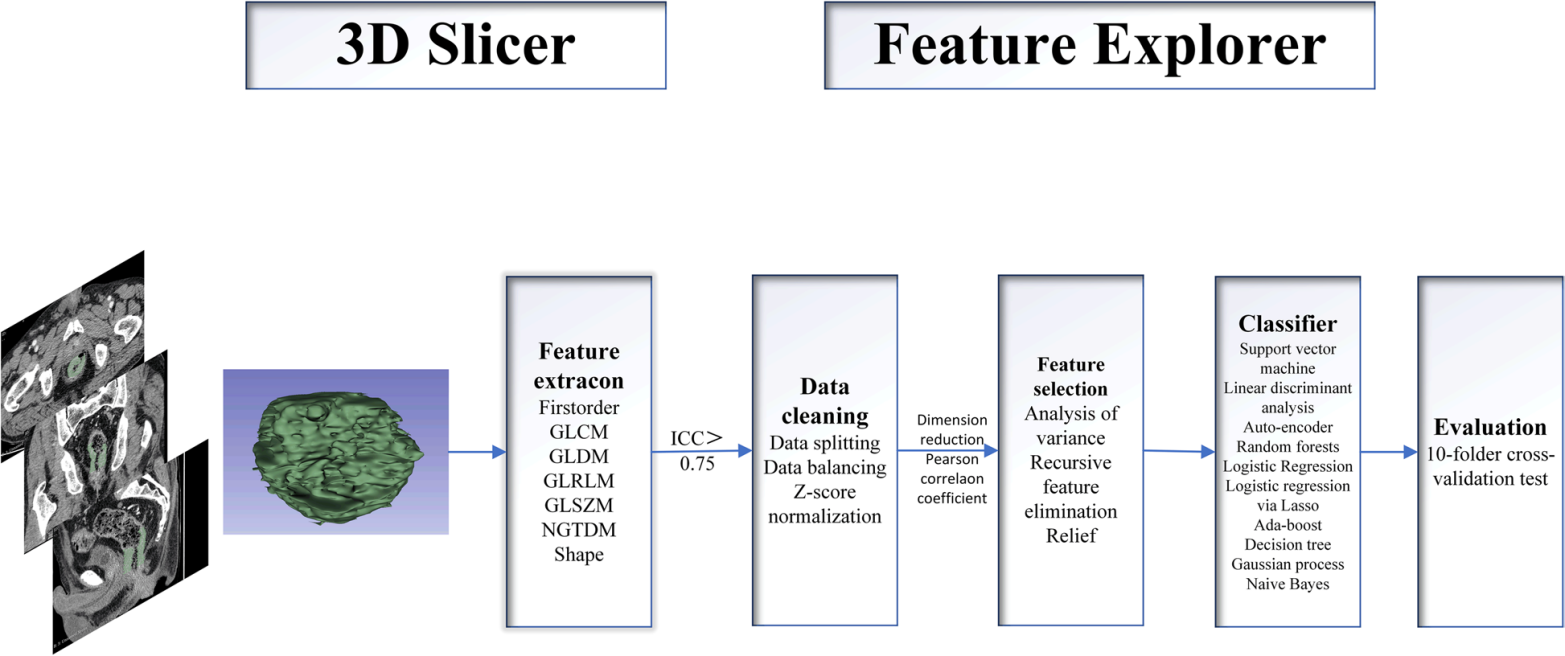
The schematic diagram of the entire radiomics and machine learning pipeline for three cohorts of patients

A tenfold cross-validation test was employed to evaluate the results. The dataset was divided into ten equal parts, with nine parts used for training and one part for validation in each iteration. The average of the results obtained from the ten iterations was used as an estimate of the algorithm’s accuracy. Accuracy, sensitivity, specificity, positive predictive value (PPV), and negative predictive value (NPV) were calculated based on the optimal cut-off value determined from the most approximate entry index. The area under the receiver operating characteristic curve (AUC) was calculated for each test condition to assess the classification performance (Fig. 2).

### Statistical analysis

SPass 26.0 was used for statistical analysis. For quantitative data, descriptive statistics such as mean ± standard deviation were used for description. For count data and ordinal data, the percentage method was used for description.

## Results

### Clinical characteristics

This study included a total of 272 consecutive patients with colorectal cancer who underwent CT examination at two hospitals from January 2016 to December 2022. Five patients were excluded due to inadequate image quality, and three patients were excluded because they could not be recognized by the 3D-slicer software. Ultimately, 244 patients were enrolled in the study. Table 1 presents the clinical data for the three cohorts of patients.

**Table 1:** The clinical data of three cohorts

Characteristics	Training cohort (*n* = 113)	Internal testing cohort (*n* = 49)	External testing cohort 1 (*n* = 82)
Age (mean ± SD)	60.12 ± 12.01	60.57 ± 11.05	66.8 ± 10.7
Sex			
Male	67 (59.3)	63.3 (64.7)	50 (61.0)
Female	46 (40.7)	36.7 (35.3)	32 (39.0)
BMI	22.79 ± 2.79	23.34 ± 3.75	22.68 ± 2.83
Pathological T staging			
T1	5 (4.4)	2 (4.1)	0 (0.0)
T2	34 (30.1)	9 (18.4)	9 (11.0)
T3	44 (39.0	25 (51.0)	69 (84.1)
T4	30 (26.5)	13 (26.5)	4 (4.9)
Pathological N staging			
N0	55 (48.7)	24 (49.0)	27 (32.9)
N1	46 (40.7)	19 (38.8)	30 (36.6)
N2	12 (10.6)	6 (12.2)	25 (30.5)
Pathological M staging			
M0	99 (87.6)	43 (87.8)	78 (95.1)
M1	14 (12.4)	6 (12.2)	4 (4.9)
Perineural invasion			
Present	61 (54.0))	27 (55.0)	52 (63.4)
Absent	52 (46.0)	22 (45.0)	30 (36.6)

*SD* Standard deviation

### Performance of the machine learning models

**Fig. 3:**
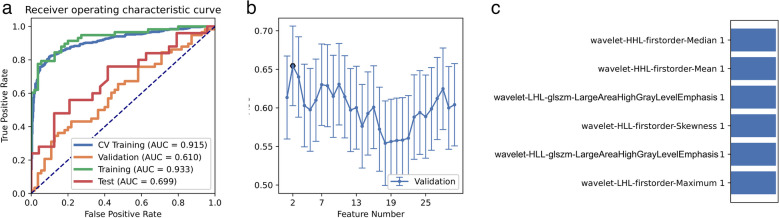
For the model performance generated by RFE, CV Train represents the average result of the K-1 folder training data set in K-folder cross-validation. CV validation represents the average result of the 1-folder dataset of the training set in K-folder cross-validation, Train represents the result by all training sets, and Test represents the result of the test set. **a** Receiver operating characteristic (ROC) curves of the model with different data sets. **b** Feature Explorer (FAE) software proposes candidate feature models according to the “one standard error” rule. **c** Features selected in diagnosis

A total of 162 patients were included in unit 1, with 83 positive and 79 negative cases. To address the impact of data imbalance on classifier filtering and eliminate imbalances in the training dataset, we promoted samples by randomly repeating cases to balance the positive and negative samples. We compared the area under the curve (AUC) of all pipelines in the validation dataset using FAE and found that pipelines using random forest feature selection and Gaussian process classifiers achieved the highest AUC of 0.610 and accuracy of 0.602 with six selected features. The resulting model achieved an AUC of 0.699 and an accuracy of 0.653 on the test dataset. Clinical statistics are presented in Table 2, while the ROC curve and selected features for diagnosis are displayed in Fig. 3.

**Table 2:** Clinical statistics in the diagnosis

Statistics value	Statistics value
Accuracy	0.6531
AUC	0.6992
AUC 95% CIs	[0.5508–0.8475]
NPV	0.6061
PPV	0.7500
Sensitivity	0.6800
Specificity	0.8333

**Fig. 4:**
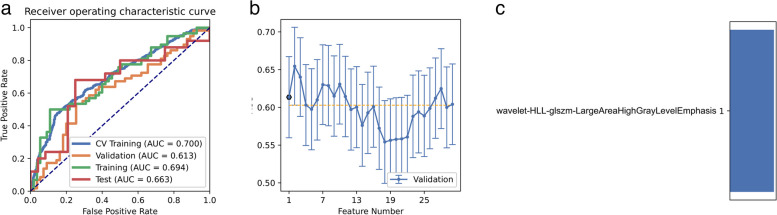
Model performance generated by RFE. **a** Receiver operating characteristic (ROC) curves of the model with different data sets. **b** Feature Explorer (FAE) software proposes candidate feature models according to the “one standard error” rule. **c** Features selected in diagnosis

While employing the RFE feature selection and Gaussian process classifier pipeline with “standard error” rules, we discovered that a model based on a single feature (wavelet-HLL-GLSZM-LargeAreaHighGrayLevelEmphasis) achieved the highest AUC of 0.614 and accuracy of 0.637 on the validation dataset. On the test dataset, this model achieved an AUC of 0.663 and accuracy of 0.610 (Fig. 4).

**Fig. 5:**
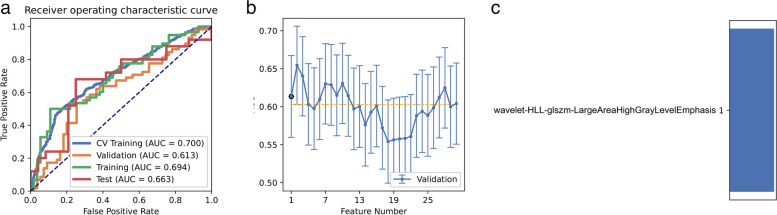
Model performance generated by ANOVA. **a** Receiver operating characteristic (ROC) curves of the model with different data sets. **b** Feature Explorer (FAE) software proposes candidate feature models according to the “one standard error” rule. **c** Features selected in diagnosis

By employing ANOVA feature selection, a pipeline utilizing GP classifiers achieved the highest AUC of 0.611 and accuracy of 0.655 on the validated dataset using a model with one feature. On the test dataset, this model achieved an AUC of 0.663 and accuracy of 0.610. The clinical statistics for the diagnosis can be found in Table 3. The ROC curve and selected features for the diagnosis are depicted in Fig. 5. Notably, when applying a “standard error” rule, we discovered that the model based on the feature (wavelet-HLL-GLSZM-LargeAreaHighGrayLevelEmphasis) yielded the same result in FAE.

**Table 3:** Clinical statistics in the diagnosis

Statistics value	Statistics value
Accuracy	0.6102
AUC	0.6633
AUC 95% CIs	[0.5042–0.8224]
NPV	0.6000
PPV	0.6455
Sensitivity	0.6400
Specificity	0.7917

For feature selector Relief, we did not get the desired result.

### External test set performance

**Fig. 6:**
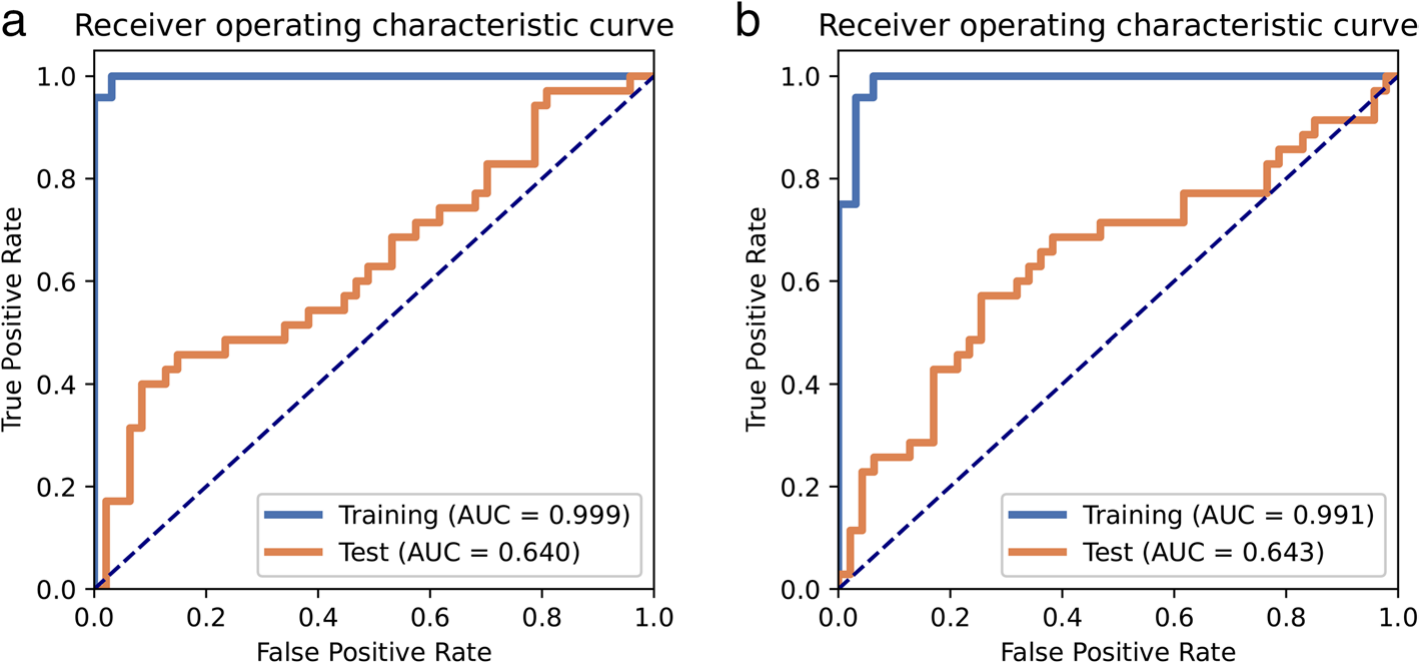
RFE-generated model performance, receiver operating characteristic (ROC) curves of the model under different data sets. **a** The ROC curve was evaluated in center 2 using RFE feature selection and the GP classifier. **b** The ROC curve was verified in center 2 using ANOVA feature selection and the GP classifier

To further assess the model’s performance, an additional 82 patients from center 2 were included. The data processing for center 2 followed the same procedures as center 1. We conducted tests on an external validation dataset using RFE feature selection and GP classifier. The model achieved an AUC of 0.640 and accuracy of 0.634 on the test dataset. Similarly, when utilizing ANOVA feature selection and GP classifier on the external validation dataset, the model attained an AUC of 0.643 and accuracy of 0.634. The ROC curve can be seen in Fig. 6.

## Discussion

This study aimed to explore the potential value of CT radiomics features in distinguishing the presence of PNI in colorectal cancer utilizing various advanced ML algorithms. The main findings are summarized as follows: (1) the pipeline incorporating RFE, ANOVA feature selection, and GP classifier exhibited stable AUC results and had the ability to achieve differential diagnosis efficiency; (2) AUC results under RFE feature selection and GP classifier were found to be higher compared to ANOVA; (3) the optimal diagnostic parameter for distinguishing PNI in CRC was found to be wavelet-LHL-glszm-LargeAreaHighGrayLevelEmphasis.

Radiomics enables the capture of microscopic tumor heterogeneity by extracting texture features from images, with 3D ROI radiomics being preferred over 2D radiomics [34, 35]. Additionally, the inclusion of multi-center data can enhance the stability and reliability of our research findings. Therefore, we extracted radiomics features based on multi-center 3D ROI and evaluated the significance of each channel in distinguishing between PNI-positive and PNI-negative cases in CRC using ML methods. The results of our study demonstrate that the ML method based on multi-center data accurately identifies the presence of PNI in CRC.

Recent studies have revealed that perineural invasion in cancer is not simply the spread of cancer cells along the connective tissue that covers nerves. Rather, it is a complex interaction between various neurotrophic and chemotactic factors present in the microenvironment surrounding cancer cells [6, 31]. PNI can lead to tumor invasion, local recurrence, and metastasis, ultimately resulting in poor prognosis. Despite its severity as an independent risk predictor for rectal cancer, PNI is challenging to determine preoperatively. Evidence from systematic reviews, meta-analyses, and other studies indicates that patients with PNI have significantly worse prognoses [14, 36–39]. In patients with resectable stage IV CRC undergoing curative surgery, PNI has been reported as a prognostic factor for survival and recurrence [40]. The prognostic significance is more pronounced in stage II and III than in stage IV. Studies have found that the order of worsening overall survival for patients is: PNI-negative stage II, PNI-positive stage II/PNI-negative stage III, and PNI-positive stage III. Therefore, it is recommended to subdivide stage II and III patients based on PNI status to provide personalized adjuvant therapy options [38]. It is worth noting that postoperative chemotherapy did not improve the 5-year disease-free survival (DFS) in PNI-negative tumor patients with stage II colon cancer. Conversely, the 5-year DFS of the PNI-positive chemotherapy group was higher. Hence, accurate prediction of PNI status is vital in evaluating the prognosis of CRC patients. Currently, the only way to determine PNI status is through pathological examination of surgical specimens. Preoperative prediction of PNI can help in the development of personalized treatment plans [18].

In this study, two feature selection methods were employed to distinguish between PNI-positive and PNI-negative cases. The parameters used mainly included first-order statistics (such as maximum, skewness, mean, median) and GLSZM (LargeAreaHighGrayLevelEmphasis) based on wavelet transform. These parameters describe the distribution of single voxel values, the statistical relationships between voxels with similar or different contrasts, and the texture frequency component data extracted from the energy calculated in the channel. Among these parameters, Wavelet-LHL-GLSZM-LargeAreaHighGrayLevelEmphasis demonstrated the highest diagnostic efficiency, indicating that the second-order parameter, LargeAreaHighGrayLevelEmphasis based on wavelet transform, is more sensitive to nerve invasion in CRC. Therefore, we speculate that the effectiveness of Wavelet-LHL-GLSZM-LargeAreaHighGrayLevelEmphasis stems from its ability to reflect the complex microstructure within the tumor and the heterogeneity of the entire tumor.

The RFE feature selection method addresses this issue by automatically removing irrelevant features and retaining the most important ones from the current feature set. This process is repeated recursively on the reduced set until the desired number of features is reached. Gaussian Processes for Machine Learning (GPML) is a general supervised learning method primarily designed for solving regression problems [33]. In our study, we utilized RFE feature selection and GP classifiers in a 10-fold cross-validation to enhance their performance on high-dimensional datasets. The results showed an accuracy of 0.653, sensitivity of 0.600, specificity of 0.833, and AUC of 0.699. Furthermore, when we applied the RFE feature selector and GP classifier on the validation dataset, the AUC value was 0.640. These findings demonstrate the feasibility and stability of the ML-based approach in identifying PNI in CRC.

Previous studies have confirmed the correlation between CT radiomics features and PNI [27]. Guo et al. [24] discovered a significant association between CT radiomics features, based on logistic regression and gray level co-occurrence matrix (GLCM), with PNI-positive patients. Chen et al. [23], using the least absolute shrinkage and selection operator (lasso) method, identified Maximum2DdiameterRow as the shape feature with the highest correlation coefficient with PNI. Liu et al. [41] reported that GLSZM, GLRLM, and first-order features were significantly correlated with PNI, and their CT radiomics model successfully predicted the PNI status of CRC. In our study, we compared three feature selection methods and 10 classifiers using FAE and found that first-order features and GLSZM were correlated with PNI. Among them, LargeAreaHighGrayLevelEmphasis based on GLSZM exhibited the highest performance in distinguishing CRC PNI. It is evident that ML based on CT radiomics features can capture the heterogeneity of CRC and predict PNI status; however, there is no consensus on the optimal feature selection methods and classifiers across studies. Further multicenter studies with larger sample sizes are necessary to reach a consensus on the specific radiomics features correlated with PNI.

There are some limitations to this study. First, potential selection bias is inevitable due to the retrospective nature of the study. Secondly, although the machine learning classifier shows promise as a predictor, the small sample size in this study may somewhat compromise the credibility of the evaluation results. Thus, increasing the sample size at each center could make the results more robust. Finally, manual mapping of ROI can be time-consuming, and there may be specific errors in the complete delineation of tumor boundaries. Therefore, further research is required to explore the potential of deep learning for automatic segmentation of lesions.

In conclusion, the model demonstrated a certain degree of discriminative and calibration ability. The ML model based on CT-based radiomics developed in this study can identify the PNI status of CRC, indicating a correlation between radiomic features of CECT images and PNI status in CRC. This model may serve as a non-invasive biomarker for preoperative assessment of PNI status in patients with colorectal cancer, as it can predict PNI in the majority of CRC patients and aid in the development of more personalized treatment plans.

## Conclusion

This study explores the feasibility of integrating artificial intelligence with radiological features to identify perineural invasion in colorectal cancer. Given its non-invasive nature, this method holds broad application prospects in clinical medicine.

### Supplementary Information

**Additional file 1: Supplementary Table A1.** Detailed CT scan parameters.

## Data Availability

The datasets used or analyzed during the current study are available from the corresponding author upon reasonable request.

## Abbreviations

ANOVA: Analysis of variance
CRC: Colorectal cancer
CT: Computed tomography
FAE: Feature Explorer software
GLRLM: Gray-level run length matrix
GLSZM: Gray-level size zone matrix
GP: Gaussian processes
ML: Machine learning
MRI: Magnetic resonance imaging
NPV: Negative predictive value
PCC: Pearson correlation coefficient
PNI: Peripheral nerve invasion
PPV: Positive predictive value
RFE: Random forests
ROC: Receiver operating characteristic
ROI: Region of interest
SVM: Support vector machines

## References

[1] Lotfollahzadeh S, Recio-Boiles A, Cagir B. Colon Cancer. StatPearls. Treasure Island (FL) with ineligible companies. Disclosure: Alejandro Recio-Boiles declares no relevant financial relationships with ineligible companies. Disclosure: Burt Cagir declares no relevant financial relationships with ineligible companies.: StatPearls Publishing Copyright © 2023, StatPearls Publishing LLC.; 2023.

[2] Sung H, Ferlay J, Siegel RL (2021). Global cancer statistics 2020: GLOBOCAN estimates of incidence and mortality worldwide for 36 cancers in 185 countries. CA Cancer J Clin.

[3] Zhang XY, Wang S, Li XT (2018). MRI of extramural venous invasion in locally advanced rectal cancer: relationship to tumor recurrence and overall survival. Radiology..

[4] Lugli A, Zlobec I (2020). The battle for prognosis at the invasive front of colorectal cancer. EBioMedicine.

[5] Benson AB, Venook AP, Al-Hawary MM (2018). Rectal cancer, version 2.2018, NCCN clinical practice guidelines in oncology. J Natl Compr Cancer Netw.

[6] Liebig C, Ayala G, Wilks JA (2009). Perineural invasion in cancer: a review of the literature. Cancer.

[7] Maguire A, Sheahan K (2014). Controversies in the pathological assessment of colorectal cancer. World J Gastroenterol.

[8] Huang CM, Huang CW, Huang MY (2014). Coexistence of perineural invasion and lymph node metastases is a poor prognostic factor in patients with locally advanced rectal cancer after preoperative chemoradiotherapy followed by radical resection and adjuvant chemotherapy. Med Principles Pract.

[9] Dhadda AS, Bessell EM, Scholefield J (2014). Mandard tumour regression grade, perineural invasion, circumferential resection margin and post-chemoradiation nodal status strongly predict outcome in locally advanced rectal cancer treated with preoperative chemoradiotherapy. Clin Oncol (R Coll Radiol).

[10] Knijn N, Mogk SC, Teerenstra S (2016). Perineural invasion is a strong prognostic factor in colorectal cancer: a systematic review. Am J Surg Pathol.

[11] Poeschl EM, Pollheimer MJ, Kornprat P (2010). Perineural invasion: correlation with aggressive phenotype and independent prognostic variable in both colon and rectum cancer. J Clin Oncol.

[12] Al-Sukhni E, Attwood K, Gabriel EM (2017). Lymphovascular and perineural invasion are associated with poor prognostic features and outcomes in colorectal cancer: a retrospective cohort study. Int J Surg.

[13] Song JH, Yu M, Kang KM (2019). Significance of perineural and lymphovascular invasion in locally advanced rectal cancer treated by preoperative chemoradiotherapy and radical surgery: can perineural invasion be an indication of adjuvant chemotherapy?. Radiother Oncol.

[14] Liebig C, Ayala G, Wilks J (2009). Perineural invasion is an independent predictor of outcome in colorectal cancer. J Clin Oncol.

[15] Borschitz T, Gockel I, Kiesslich R (2008). Oncological outcome after local excision of rectal carcinomas. Ann Surg Oncol.

[16] Peng J, Chen W, Sheng W (2011). Oncological outcome of T1 rectal cancer undergoing standard resection and local excision. Colorectal Dis.

[17] Cienfuegos JA, Martínez P, Baixauli J (2017). Perineural invasion is a major prognostic and predictive factor of response to adjuvant chemotherapy in stage I-II colon cancer. Ann Surg Oncol.

[18] Cienfuegos JA, Rotellar F, Baixauli J (2015). Impact of perineural and lymphovascular invasion on oncological outcomes in rectal cancer treated with neoadjuvant chemoradiotherapy and surgery. Ann Surg Oncol.

[19] Giger OT, Comtesse SC, Lugli A (2012). Intra-tumoral budding in preoperative biopsy specimens predicts lymph node and distant metastasis in patients with colorectal cancer. Modern Pathol.

[20] Nikberg M, Chabok A, Letocha H (2016). Lymphovascular and perineural invasion in stage II rectal cancer: a report from the Swedish colorectal cancer registry. Acta Oncol.

[21] Lino-Silva LS, Salcedo-Hernández RA, España-Ferrufino A (2017). Extramural perineural invasion in pT3 and pT4 rectal adenocarcinoma as prognostic factor after preoperative chemoradiotherapy. Hum Pathol.

[22] Chablani P, Nguyen P, Pan X (2017). Perineural invasion predicts for distant metastasis in locally advanced rectal cancer treated with neoadjuvant chemoradiation and surgery. Am J Clin Oncol..

[23] Chen Q, Cui Y, Xue T (2022). Computed tomography-based radiomics nomogram for the preoperative prediction of perineural invasion in colorectal cancer: a multicentre study. Abdom Radiol (NY).

[24] Guo Y, Wang Q, Guo Y (2021). Preoperative prediction of perineural invasion with multi-modality radiomics in rectal cancer. Sci Rep.

[25] Li M, Jin YM, Zhang YC (2021). Radiomics for predicting perineural invasion status in rectal cancer. World J Gastroenterol.

[26] Li Y, Eresen A (2020). Preoperative prediction of perineural invasion and KRAS mutation in colon cancer using machine learning. J Cancer Res Clin Oncol.

[27] Huang Y, He L, Dong D et al (2018) Individualized prediction of perineural invasion in colorectal cancer: development and validation of a radiomics prediction model. Chin J Cancer Res 30:40–50. 10.21147/j.issn.1000-9604.2018.01.05 10.21147/j.issn.1000-9604.2018.01.05 PMC5842232 29545718

[28] Huang J, Chen Y, Zhang Y (2022). Comparison of clinical-computed tomography model with 2D and 3D radiomics models to predict occult peritoneal metastases in advanced gastric cancer. Abdom Radiol (NY).

[29] Xu L, Yang P, Yen EA (2019). A multi-organ cancer study of the classification performance using 2D and 3D image features in radiomics analysis. Phys Med Biol.

[30] Chen C, Qin Y, Chen H (2022). Machine learning to differentiate small round cell malignant tumors and non-small round cell malignant tumors of the nasal and paranasal sinuses using apparent diffusion coefficient values. Eur Radiol.

[31] Chen J, Chen Y, Zheng D (2021). Pretreatment MR-based radiomics nomogram as potential imaging biomarker for individualized assessment of perineural invasion status in rectal cancer. Abdom Radiol (NY).

[32] Bakst RL, Wong RJ (2016). Mechanisms of perineural invasion. J Neurol Surg Part B Skull Base.

[33] Song Y, Zhang J, Zhang YD (2020). FeAture Explorer (FAE): a tool for developing and comparing radiomics models. PLoS One.

[34] Wan Q, Zhou J, Xia X (2021). Diagnostic performance of 2D and 3D T2WI-based radiomics features with machine learning algorithms to distinguish solid solitary pulmonary lesion. Front Oncol.

[35] Xie XJ, Liu SY, Chen JY (2021). Development of unenhanced CT-based imaging signature for BAP1 mutation status prediction in malignant pleural mesothelioma: consideration of 2D and 3D segmentation. Lung Cancer.

[36] van Wyk HC, Going J, Horgan P (2017). The role of perineural invasion in predicting survival in patients with primary operable colorectal cancer: a systematic review. Crit Rev Oncol Hematol.

[37] Yang Y, Huang X, Sun J (2015). Prognostic value of perineural invasion in colorectal cancer: a meta-analysis. J Gastrointest Surg.

[38] Zhou Y, Wang H, Gong H (2015). Clinical significance of perineural invasion in stages II and III colorectal cancer. Pathol Res Pract.

[39] Mayo E, Llanos AA, Yi X (2016). Prognostic value of tumour deposit and perineural invasion status in colorectal cancer patients: a SEER-based population study. Histopathology.

[40] Yun HR, Lee WY, Lee WS (2007). The prognostic factors of stage IV colorectal cancer and assessment of proper treatment according to the patient’s status. Int J Colorectal Dis.

[41] Liu J, Sun L, Zhao X (2023). Development and validation of a combined nomogram for predicting perineural invasion status in rectal cancer via computed tomography-based radiomics. J Cancer Res Ther.

