# Supplementary material for: The value of machine learning based on CT radiomics in the preoperative identification of peripheral nerve invasion in colorectal cancer: a two-center study
Source: Insights Imaging. 2024 Apr 5;15:101. doi: 10.1186/s13244-024-01664-1 (PMC10997560; doi:10.1186/s13244-024-01664-1)
Supplement: Supplementary file 1 — Additional file 1: Supplementary Table A1. Detailed CT scan parameters. [file 13244_2024_1664_MOESM1_ESM.pdf]

The value of machine learning based on CT radiomics in the preoperative identification of peripheral nerve invasion in colorectal cancer: A Two-Center Study

ELECTRONIC SUPPLEMENTARY MATERIAL

Parameter	Center 1	Center 2
Scanners and manufacturers	64-slice spiral CT (SIEMENS, Germany)	128-slice spiral CT (PHILIPS Brilliance, Holland)
Tube potential	120 kVp	120 kVp
Tube current	200 ~ 300 mAs	200 ~ 300 mA
Rotation time	0.50 s	0.50 s
Detector collimation	64×0.6 mm	64×0.6 mm
Contrast agent type	Iohexol (Yang zi jiang Pharmaceutical Group, Jiangsu, China)	Ioversol (Heng rui Medicine, Jiangsu, China)
Contrast agent infusion rate	2.5-3.5 ml/s	3.5 ml/s
Contrast agent concentration	350 mg/ml	320 mg/ml
Contrast agent dosage	1.2 ml/kg	1.0-1.5 ml/kg
Field of view	350×350 mm	350×350 mm
Intelligent tracking trigger scan threshold (Aorta)	100 HU	100 HU
Arterial phase	10 s after the intelligent tracking triggers the scanning threshold	10 s after the intelligent tracking triggers the scanning threshold
Venous phase	60-70 s after injection of contrast agent	60-70 s after injection of contrast agent
Delayed	180 s after injection of contrast agent	180 s after injection of contrast agent
Thickness	1.0 mm	1.0 mm
Matrix	512×512	512×512
Reconstruction interval	1.0 mm	1.0 mm

CT parameters of the two centers

Insights Imaging (2024) Liu NJ, Liu MS, Tian W, et al.
